# Identification of the hub genes RUNX2 and FN1 in gastric cancer

**DOI:** 10.1515/med-2020-0405

**Published:** 2020-05-30

**Authors:** Chao Han, Lei Jin, Xuemei Ma, Qin Hao, Huajun Lin, Zhongtao Zhang

**Affiliations:** Department of General Surgery, Beijing Friendship Hospital, Capital Medical University, Beijing, 100050, China

**Keywords:** bioinformatics analysis, FN1, gastric cancer, prognosis, RUNX2

## Abstract

**Background:**

This study identified key genes in gastric cancer (GC) based on the mRNA microarray GSE19826 from the Gene Expression Omnibus (GEO) database and preliminarily explored the relationships among the key genes.

**Methods:**

Differentially expressed genes (DEGs) were obtained using the GEO2R tool. The functions and pathway enrichment of the DEGs were analyzed using the Enrichr database. Protein–protein interactions (PPIs) were established by STRING. A lentiviral vector was constructed to silence RUNX2 expression in MGC-803 cells. The expression levels of RUNX2 and FN1 were measured. The influences of RUNX2 and FN1 on overall survival (OS) were determined using the Kaplan–Meier plotter online tool.

**Results:**

In total, 69 upregulated and 65 downregulated genes were identified. Based on the PPI network of the DEGs, 20 genes were considered hub genes. RUNX2 silencing significantly downregulated the FN1 expression in MGC-803 cells. High expression of RUNX2 and low expression of FN1 were associated with long survival time in diffuse, poorly differentiated, and lymph node-positive GC.

**Conclusion:**

High RUNX2 and FN1 expression were associated with poor OS in patients with GC. RUNX2 can negatively regulate the secretion of FN1, and both genes may serve as promising targets for GC treatment.

## 1 Introduction

Gastric cancer (GC) is a common malignant digestive tract tumor with a poor prognosis. GC incidence ranks fifth globally for mortality of all malignancies and third for cancer-related mortality of all malignancies, and the majority of GC cases occur in the developing countries [1,2,3]. In China, GC is one of the most common malignancies and was the second leading cause of cancer-related death in males and the fifth leading cause of cancer-related death in females in 2014 [4]. In the Western world, the majority of patients with GC are diagnosed at an advanced stage, so radical surgical treatment is no longer possible at the time of diagnosis, leading to poor prognosis. The expected 5-year survival for patients is approximately 26% in Western countries. Surgical resection is still the only curative therapy for nonmetastatic GC, while neoadjuvant chemotherapy, adjuvant chemotherapy chemoradiotherapy, and targeted therapy can reduce recurrence and prolong survival [5,6,7].

Complex signaling molecules lead to poor prognosis by affecting the occurrence and development of tumors [8]. Currently, the application of high-throughput platforms in gene expression analysis is becoming more valuable in many aspects of clinical research [9,10,11]. Through second-generation sequencing technology, hundreds of differentially expressed genes (DEGs) have been discovered and are involved in different tumor cell biological processes; the relationships between these genes must be very complicated. RUNX2 is an evolutionarily conserved regulator of cell fate, which contains a highly conserved runt domain. RUNX2 can recognize and directly bind to the consensus sequences (TGTGGT or ACCACA) and mediate transcriptional activation or repression of target genes [12]. Boregowda et al. showed that RUNX2 is overexpressed in melanoma cells and mediates their migration and invasion by affecting the expression of focal adhesion kinase [13]; Cao et al. reported that RUNX2 was associated with EMT by regulating galectin-3 expression in hepatocellular carcinoma [14]. RUNX2 has been shown to be involved in many molecular mechanisms of tumor progression [15,16,17]. Although Guo et al. found that the transcription factor RUNX2 upregulates the chemokine receptor CXCR4 to promote the invasive and metastatic potential of human GC [18], research on RUNX2 in GC has been rarely been conducted. The specific mechanism underlying the participation of RUNX2 in GC pathogenesis remains unclear. Furthermore, studies have shown that FN1 is involved in tumor invasion and metastasis [19,20]. However, no reports on the relationship between RUNX2 and FN1 in GC have been reported until now.

In this study, we aimed to screen the hub genes (genes with a high degree of connections in the gene expression network, which do not involve betweenness) in the development of GC. By constructing a PPI network and using cytoHubba, we found that RUNX2 and FN1 are hub genes in DEGs, that FN1 ranks number 1, and that these genes are connected. RUNX2 is a transcription factor, and we found that FN1 may be a target gene of the transcription factor RUNX2 through the online website Harmonizome (http://amp.pharm.mssm.edu/Harmonizome/). Therefore, we decided to inhibit the expression of RUNX2 in MGC803 cells to observe the changes in FN1 by transfection with lentiviral vectors. Finally, we obtained the prognostic value (the association of the protein level with the survival time of patients with GC) of RUNX2 and FN1 in GC from the KM plotter (http://kmplot.com/analysis/).

## 2 Materials and methods

### 2.1 Microarray data and identification of DEGs

The dataset GSE19826 is based on the platform of GPL 570 (Affymetrix Human Genome U133 Plus 2.0 Array) and contains 12 GC tissue, 12 noncancer tissue, and three normal gastric tissue samples from the National Center for Biotechnology Information (NCBI) Gene Expression Omnibus (GEO) database (https://www.ncbi.nlm.nih.gov/geo). GEO2R, supplied by NCBI (https://www.ncbi.nlm.nih.gov/geo/geo2r/), is an interactive web tool that was used to identify the DEGs between GC tissue samples and noncancerous tissue samples. GEO2R performs comparisons on original submitter-supplied processed data tables using the GEOquery and limma R packages from the Bioconductor project [21]. In the current study, genes with |log FC| ≥ 1.5 and *p* < 0.05 (adjusted by the false discovery rate) were regarded as DEGs [22]. Also, the GSE79973 dataset was used to validate the findings.

### 2.2 Gene ontology (GO) and Kyoto encyclopedia of genes and genomes (KEGG)

To elucidate the potential biological processes, we performed GO enrichment analysis utilizing the clusterProfiler package in R software. KEGG pathway enrichment analysis was also carried out by the clusterProfiler package in R software to identify promising signaling pathways correlated with the DEGs. An adjusted *p*-value < 0.05 was considered significant.

### 2.3 Protein–protein interaction (PPI) network building

STRING database version 10.5 (http://string-db.org) was applied to evaluate the PPI information. DEGs were uploaded into STRING, a confidence score >0.9 defined them as significant [23], and then, the PPI network was constructed. We downloaded the resulting data in a table in a tab-separating values format. The data obtained were uploaded into Cytoscape software, which was used to construct PPI relationship subnetworks. The cytoHubba plug-in was used to identify the hub genes. A hub gene is defined as a gene with the highest degree of connectivity in the hub module [23]. The hub genes showed a strong association with other node proteins (more than 10) [23].

### 2.4 Cell culture

The human GC cell line MGC-803 was obtained from the Department of General Surgery, Beijing Friendship Hospital (Beijing, China). The cells were maintained in the Dulbecco’s modified Eagle’s medium (HyClone, Logan, UT, USA) containing 10% FBS (HyClone, Logan, UT, USA), 100 mg/mL penicillin, and 100 mg/mL streptomycin. Cells were cultured at 37℃ in a humidified atmosphere with 5% CO_2_.

### 2.5 Gene transfection

We synthesized three pairs of self-complementing hairpin DNA fragments targeting RUNX2 mRNA (shRUNX2#1, ACCATAACCGTCTTCACAAAT; shRUNX2#2, GCACGCTATTAAATCCAAATT; and shRUNX2#3, AGGTTCAACGATCTGAGATTT) and scramble DNA (CCTAAGGTTAAGTCGCCCTCG) and cloned them into lentiviral vectors to knockdown RUNX2 in MGC-803 cells. The packaged lentiviral particles containing shRUNX2 and scrambled shRNA were named lentivirus-shRUNX2 (shRUNX2#1, #2, and #3) and lentivirus-Scramble (Scramble_shRNA), respectively. We transfected MGC-803 cells with shRUNX2 and Scramble with an optimal MOI of 10 in the presence of 6 µg/mL polybrene [18]. Stable transfection of MGC-803 cells was observed by green fluorescent protein. We used quantitative real-time PCR (qRT-PCR) and Western blot to detect the efficiency of RUNX2 knockdown.

### 2.6 RNA extraction and qRT-PCR

Total RNA from cells was extracted using an RNA-Quick Purification Kit (ESscience Biotech, Shanghai, China). RNA (2–5 µg) was reverse transcribed in a 25-µL reaction system using the Thermoscript™ RT-PCR System kit. Primers for RUNX2, FN1, and β-actin were synthesized by TaKaRa (Dalian, China). The PCR primers for RUNX2 were 5′-CAGAGCAACGTGCTCCAAAGTC′-3′ (forward) and 5′-GAAGCGTTGCTGTCGGTTCA-3′ (reverse). The PCR primers for FN1 were 5′-GTTCGGGAGGAGGTTGTTACC-3′ (forward) and 5′-GAGTCATCTGTAGGCTGGTTTAGG-3′ (reverse). The primers for β-actin were 5′-TCATGAAGTGTGACGTTGACATCCGT-3′ (forward) and 5′-CCTAGAAGCATTTGCGGTGCACGATG-3′ (reverse). RT-PCR was carried out using 2× SYBR Green qPCR Mix to detect the contents of RUNX2, FN1, and β-actin. Then, RT-PCR was performed according to the recommended conditions: predenaturation at 95℃ for 10 min, 95℃ for 15 s, 60℃ for 20 s, 72℃ for 20 s, and 44 cycles of 95℃ for 15 s, 60℃ for 15 s, and 95℃ for 15 s. The specificity of the results was ensured by the dissolution curve, and the relative quantitative 2^−ΔΔCt^ method was used for data analysis. Gene expression was normalized to β-actin expression. Each sample was repeated three times with qRT-PCR.

### 2.7 Western blot analysis

MGC-803 cells transfected with lentivirus-shRUNX2#1 were washed twice in cold PBS and then lysed with protein extraction reagent (Beyotime Institute of Biotechnology Jiangsu, China) containing protease inhibitors (Merk, USA). The protein concentration of the cell lysates was quantified using a BCA Kit (Beyotime Institute of Biotechnology Jiangsu, China), and 50 ng of protein for each sample was separated by SDS-PAGE on 10% gels and then transferred to PVDF membranes (Millipore, USA). Following blocking with 5% skim milk suspended in Tris-buffered saline containing Tween-20 (TBST) at room temperature for 1 h, the membranes were incubated overnight at 4°C with primary antibodies as follows: anti-FN1 (1:5,000; GeneTex, Zeeland, USA), anti-RUNX2 (1:1,000; ABGENT, San Diego, USA), and anti-β-actin (1:5,000; Abcam, Cambridge, UK). The membranes were then incubated with a goat anti-rabbit or anti-mouse IgG secondary antibody conjugated to horseradish peroxidase (1:1,000; Cell Signaling Technology, Inc., MA, USA) for 1 h. Chemiluminescence was detected using SuperSignal West Femto Maximum Sensitivity Substrate (ECL, Pierce) in a ChemiDoc XRS system (Bio-Rad). The density of the bands was measured using ImageJ software (USA).

### 2.8 The prognostic value of RUNX2 and FN1

The prognostic value of RUNX2 and FN1 was obtained from the KM plotter (http://kmplot.com/analysis/).

### 2.9 Statistical analysis

All statistical analyses were performed using GraphPad Prism 6.0 software (GraphPad Software, Inc., USA). Data are presented as the mean ± standard deviation. Student’s *t*-test was used to compare the differences between the two groups. Differences were considered statistically significant at a *p* value of < 0.05.

## 3 Results

### 3.1 Identification of DEGs

A total of 134 DEGs were identified from the GSE19826 dataset and selected for further analysis. There are 69 upregulated genes and 65 downregulated genes in GC tissues when compared with those in noncancer tissues (Table 1). Interestingly, the log FC value of RUNX2 was +1.87, and the log FC value of FN1 was −1.72, and a negative correlation might exist between them. The dataset GSE79973 further validated the findings (Supplementary Table 1).

**Table 1:** DEGs in the GSE19826 dataset

	Genes
Upregulated	C19orf47, NFE2L2, SMIM6, UBL3, SIRT5, METTL7A, MYRF, C8orf49, LOC101927533, MGC10814, CACNA1E, MINCR, BCL2L10, FLJ33534, SLC2A12, PLIN5, ADTRP, PTPRE, TMPRSS2, RUNX2, KRTAP2-1, HPS6, CABP1, ACSM3, GRK2, LOC101928457, PHKG2, FMO5, IL12A, FRMD1, GATA4, TCEB3, LIFR, LOC729296, RERE, RASSF6, DNAI2, TRIM50, ATP6V0E2, AKR7A3, JPH2, C11orf87, SNTG1, HYAL1, C9orf66, CNGB3, EPHA6, FAM90A2P, KIR2DS4, CCDC169, CLIC6,MYOC, LINC00161, ASB11, RDH12, LOC101928335, KLK11, PSAPL1, OR2C3, DCAF12L1, MUC5AC, FAM167A, VSIG1, GRIA4, MFSD4A, SH3GL2, DPCR1, MUC6, DPCR1
Downregulated	INHBA, HOXA13, HOXA10, COL10A1, FAP, GPR78, SALL4, COL8A1, SPP1, TNFRSF11B, ADAMTS2, SERPINH1, THBS2, ONECUT2, HOXA10, REEP6, HKDC1, COL1A1, P4HA3, SULF1, SFRP2, SFRP4, DLGAP1-AS2, PDLIM7, ADAMTS12, MFAP2, SNX8, BMP1, MGP, CLEC11A, MARVELD3, CENPF, HRH1, COL1A2, HIST1H2BJ, MIR181A2HG, TEAD4, PLA2G4C, KLHL25, PGF, THY1, C6orf1, POM121L10P, COL6A3, PRRX1, ANKH, APOE, HOXB7, APOC1, TIMP1, FN1, BGN, COL5A1, GPNMB, LINC01123, NID2, SPPL2B, COL12A1, SPARC, COL3A1, FBN1, IGFBP4, SOX4, HEYL, COL5A2

A total of 134 genes were in the common region, which contained 69 upregulated and 65 downregulated DEGs.

### 3.2 PPI network construction

**Figure 1:**
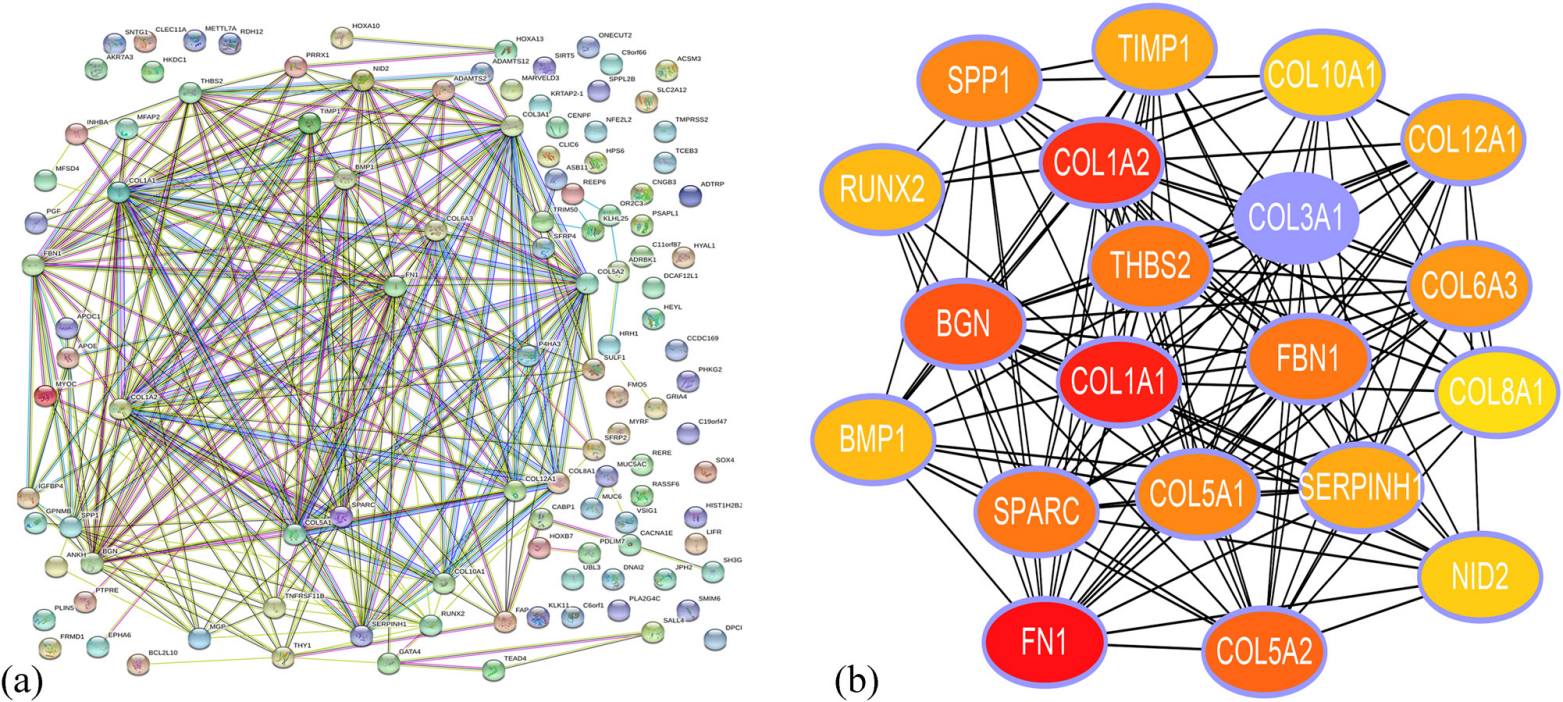
PPI analysis. (a) PPI network constructed from STRING. The network nodes represent proteins, and edges demonstrate the predicted functional associations between them. Seven different colored lines indicate the edges representing the seven types of evidence used in predicting the associations. The lines indicate the following evidence: red line – fusion; green line – neighborhood; blue line – co-occurrence; purple line – experimental; yellow line – text-mining; light blue line – database; black line – co-expression. (b) Twenty hub genes were obtained from the PPI network of DEGs using cytoHubba. The depth of the color represents the rank of the hub genes: red, blue, orange, and yellow indicate rankings in decreasing order; the darker the color, the higher the ranking is. PPI, protein–protein interactions; DEGs, differentially expressed genes.

The PPI network of DEGs consisted of 134 nodes and 235 edges, including 69 upregulated genes and 65 downregulated genes (Figure 1a). Using cytoHubba, 20 hub genes were obtained from the PPI network of DEGs: FN1, COL1A1, COL1A2, COL3A1, BGN, COL5A2, THBS2, SPARC, FBN1, COL5A1, SPP1, COL6A3, TIMP1, SERPINH1, COL12A1, RUNX2, BMP1, COL10A1, NID2, and COL8A1 (Figure 1b); these hub genes had a high degree of connectivity (Table 2). FN1 ranks No. 1 with the highest score of the 20 hub genes.

**Table 2:** The rankings of the hub genes based on connectivity

Rank	Name	Score (number of connections)
1	FN1	32
2	COL1A1	31
3	COL1A2	28
4	COL3A1	25
5	BGN	24
6	COL5A2	20
7	THBS2	20
7	SPARC	20
7	FBN1	19
10	COL5A1	19
10	SPP1	17
12	COL6A3	16
13	TIMP1	16
13	SERPINH1	16
13	COL12A1	13
16	RUNX2	13
16	BMP1	13
18	COL10A1	12
18	NID2	12
20	COL8A1	11

### 3.3 GO term and KEGG pathway enrichment analyses of DEGs

**Figure 2:**
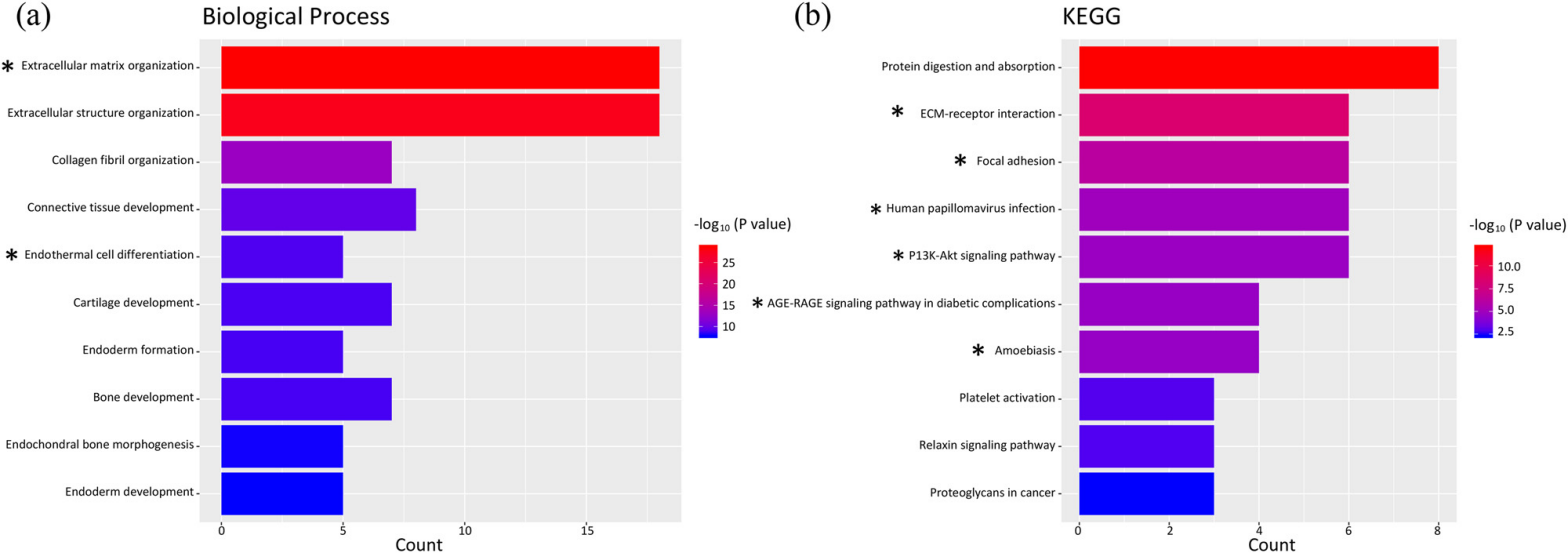
GO term and KEGG pathway enrichment analyses of the DEGs. (a) Biological process (BP) term enrichment analysis for the DEGs. (b) KEGG pathway enrichment analysis for the DEGs. GO, Gene Ontology; KEGG, Kyoto Encyclopedia of Genes and Genomes. An asterisk “*” denotes FN1-related pathways.

GO term and KEGG pathway enrichment analyses were performed using the clusterProfiler package in R software. The DEGs were mainly involved in the biological processes extracellular matrix organization, extracellular structure organization, collagen fibril organization, and connective tissue development (Figure 2a). KEGG pathway analysis showed that the DEGs were mainly enriched in protein digestion and absorption, ECM–receptor interaction, and focal adhesion (Figure 2b). FN1 is involved in an extracellular matrix organization and endodermal cell differentiation in BP term enrichment analysis and ECM–receptor interaction, focal adhesion, amoebiasis, AGE-RAGE signaling pathway in diabetic complications, human papillomavirus infection, and PI3K-Akt signaling pathway in the KEGG pathway analysis (Figure 2). RUNX2 was categorized in ossification, osteoblast differentiation, endochondral ossification, and osteoblast fate commitment in BP term enrichment analysis and parathyroid hormone synthesis, secretion, and action and transcriptional misregulation in cancer in the KEGG pathway analysis (not shown in Figure 2 due to a relatively low score).

### 3.4 Downregulation of RUNX2 increased FN1 expression in MGC803 cells

**Figure 3:**
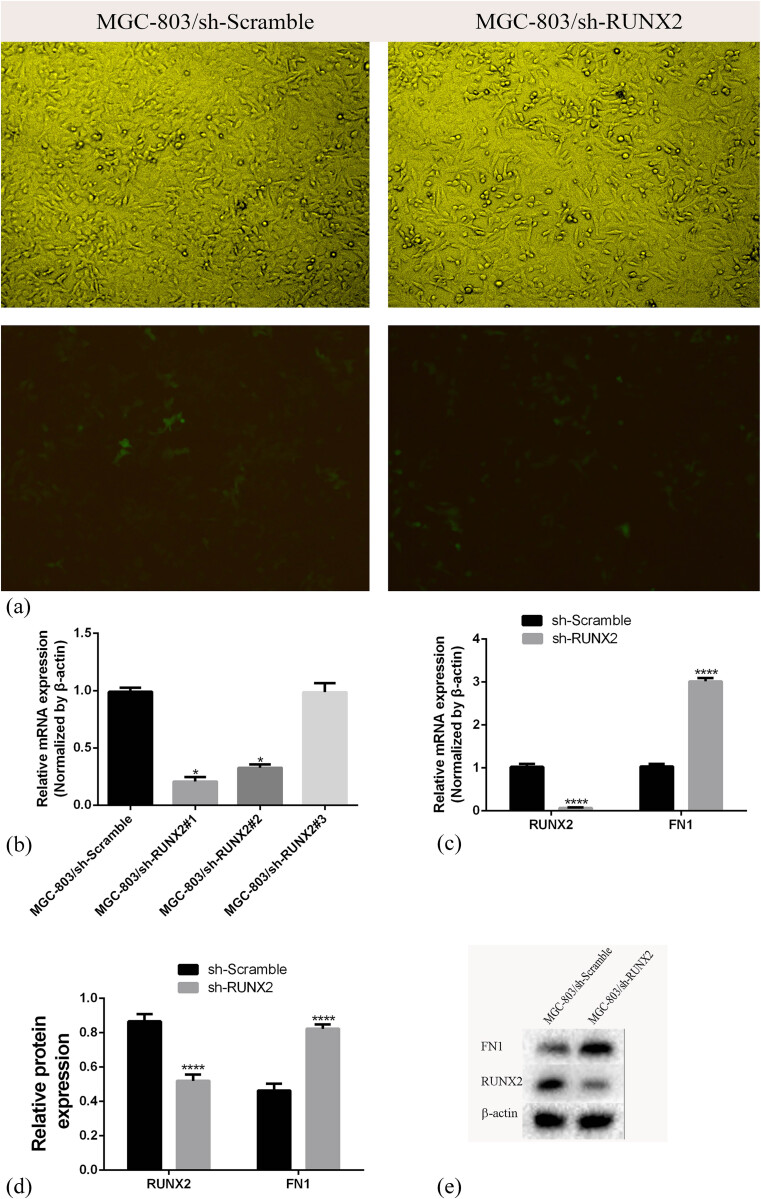
MGC-803 cells were transfected with lentivirus-shRUNX2 (MGC-803/sh-RUNX2) and lentivirus-Scramble (MGC-803/sh-NC). (a) Representative graphs of MGC-803 cells infected with the indicated lentivirus. (b) Transfection efficacy of the three lentivirus-shRUNX2 vectors. (c) RUNX2 and FN1 mRNA levels in the two groups after stable transfection. (d) and (e) RUNX2 protein expression levels after stable transfection (****p < 0.0001).

We found 20 hub genes in the GSE19826 dataset. Both RUNX2 and FN1 are hub genes, and FN1 ranks No. 1 with the highest score of the 20 hub genes; thus, we concluded that FN1 may play an important role in GC. Moreover, studies about the hub gene RUNX2 in GC are lacking, so we are very interested in its role in GC. Taken together, we decided to conduct a preliminary study of the correlation between the two. To explore the changes in FN1 in GC cells with RUNX2, we transfected MGC803 cells with shRUNX2 and Scramble with an MOI of 10 in the presence of 6 µg/mL polybrene (Figure 3a). qRT-PCR was used to test the transfection efficacy of shRUNX2#1, shRUNX2#2, and shRUNX2#3; Lentivirus-shRUNX2#1 had the best transfection efficacy; thus, we chose MGC-803/shRUNX2#1 for further experiments (Figure 3b). qRT-PCR and Western blot analyses confirmed RUNX2 downregulation in MGC803 cells (*p* < 0.0001), and the expression of FN1 in MGC803 cells increased significantly with the downregulation of RUNX2 (*p* < 0.0001) (Figure 3c–e).

### 3.5 The prognostic value of RUNX2 and FN1

**Figure 4:**
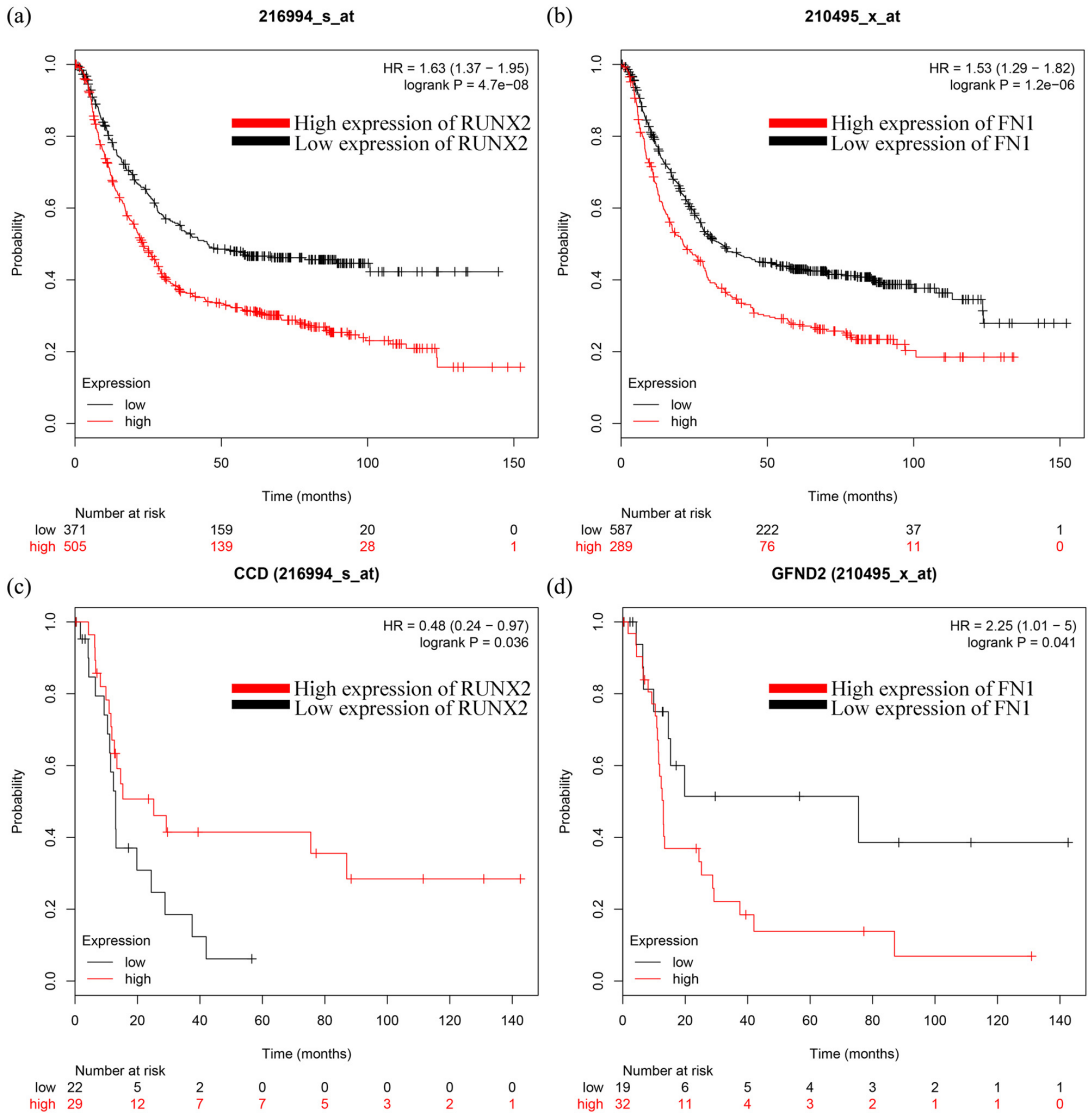
Prognostic value of RUNX2 and FN1 from the KM plotter (http://kmplot.com/analysis/). (a) Prognostic value of RUNX2. (b) Prognostic value of FN1. (c) Prognostic value of RUNX2 (diffuse, poorly differentiated patients with GC). (d) Prognostic value of FN1 (diffuse, poorly differentiated patients with GC). The desired Affymetrix IDs are valid: 216994_s_at (RUNX2), 210495_s_at (FN1). HR, hazard ratio.

The prognostic value of RUNX2 and FN1 was obtained from the KM plotter (http://kmplot.com/analysis/). According to the expression of a particular gene, the patients with GC were split into two groups with high and low expression. The overall survival (OS) of patients with GC was evaluated using a KM plot. The hazard ratio (HR) with 95% confidence intervals and log-rank *P* values are shown on the web page. As shown in Figure 4a and b, GC patients with higher expression of RUNX2 had a lower probability of survival than those with lower RUNX2 expression. However, those with higher expression of FN1 also had a lower probability of survival, which contradicted the results obtained in the cell experiments. Then, subgroup (those with diffuse, poorly differentiated, and lymph node-positive GC) analysis was performed. As shown in Figure 4c and d, the median survival of patients with higher expression of RUNX2 was significantly longer than those with lower expression, whereas patients with lower expression of FN1 had a significantly longer median survival time than those with higher expression of FN1.

## 4 Discussion

GC is still a disease with high rates of morbidity and mortality due to its high heterogeneity. Although surgery is the main treatment method, the other treatments, including chemotherapy, radiotherapy, targeted therapy, and gene therapy, are available; however, the 5-year survival rate was still less than 30% [24]. GC is a main contributor to the global burden of disability-adjusted life-years from cancer in men [25]. The burden of GC remains very high in Asia, Latin America, and Central and Eastern Europe, whereas it is no longer a common cancer in North America and most Western European countries [26]. It is essential to explore the mechanisms of GC progression to prevent its occurrence, improve treatment efficacy, and improve the survival rate of patients with GC.

In this study, 134 DEGs were screened, consisted of 69 upregulated genes and 65 downregulated genes. These genes were mainly enriched in extracellular matrix organization, collagen fibril organization, and protein complex subunit organization in biological processes. Among these DEGs, 20 genes had high degrees in the PPI network, including FN1, COL1A1, COL1A2, BGN, SPARC, and RUNX2. FN1 ranked first in the hub genes. The hub genes we found are partly the same as the hub genes found in other studies [22,27]. However, there were inconsistencies. The reason may be that multiple data sets intersected in some studies, which will inevitably lead to the loss of some hub genes [22,27].

As a member of the RUNX family, RUNX2 is identified by the runt-homology domain. In contrast to RUNX1 and RUNX3, whose mutations are closely linked to the promotion of leukemia and GC, respectively [28,29], the initial studies suggest that RUNX2 acts as a master regulator of osteoblast differentiation and bone development. During bone development, RUNX2 facilitates the differentiation of mesenchymal stem cells into osteoblast lineage cells [30]. Some studies suggest that RUNX2 has the ability to transactivate its downstream target genes, such as MMP9, MMP13, VEGF, survivin, and IL-8, which are involved in tumor progression, invasion, and metastasis [31,32,33,34,35,36]. FN1 is a member of the ligand glycoprotein family, which is widely expressed in various cell types and is involved in cell adhesion and migration [37]. As a transcription factor, the studies of RUNX2 in GC were relatively less, and we have a keen interest in it. FN1 ranks first in the DEGs of GSE19826, so we decided to conduct a preliminary exploration of the correlation between RUNX2 and FN1. We detected that the expression level of FN1 was upregulated in MGC803 cells by RT-qPCR and Western blot analyses. Therefore, we initially concluded that RUNX2 can act negatively on the FN1 gene in GC. This result seems inconsistent with the results of the prognostic value of the two genes obtained from the KM plotter. We performed a subgroup analysis of the two genes in the same datasets; to our surprise, we found that among the patients with diffuse and poorly differentiated GC, those with higher expression of RUNX2 had a significantly higher median survival time than those with lower RUNX2 expression, whereas patients with lower expression of FN1 had a significantly longer median survival time than those with higher FN1 expression (Figure 4c and d). Based on our findings, we speculate that the expression of RUNX2 in GC tissue changes with the degree of tumor malignancy, and its effect on FN1 also changes, and with a high degree of malignancy, the two genes are negatively correlated. To date, the role of RUNX2 in carcinogenesis and cancer progression remains unclear and may be tissue- and context-dependent [38]. RUNX2 may play a promoting role in invasive bone cancer [39], prostate cancer [31], pancreatic cancer [40], and GC [18]. Nevertheless, Chimge et al. showed that RUNX2 may possess tumor suppressor properties in breast cancer [41]. The overexpression of RUNX2 leads to the upregulation of Bax and increased sensitivity to apoptosis [42]. FN1 can downregulate P53 and inhibit apoptosis in colorectal cancer [43]. Considering that apoptosis is one of the mechanisms of drug resistance [44], we hypothesized that the mechanism by which RUNX2 negatively regulates FN1 may be involved in chemoresistance in GC.

This preliminary study was based on bioinformatics analyses with only single-cell experiments and with only qRT-PCR and Western blot analyses to verify the relationship between RUNX2 and FN1. The specific roles of the two hub genes in GC need to be verified by further research. In the future, more cell lines will be added, and clinical samples will be combined to further explore the mechanisms of two hub genes involved in the invasion, metastasis, and drug resistance of GC.

In summary, FN1, COL1A1, COL1A2, COL3A1, BGN, COL5A2, THBS2, SPARC, FBN1, COL5A1, SPP1, COL6A3, TIMP1, SERPINH1, COL12A1, RUNX2, BMP1, COL10A1, NID2, and COL8A1 might be hub genes of GC. The transcription factor RUNX2 may act negatively on the FN1 gene, and they might be correlated with the prognosis of GC. This study might shed new light on the correlation between RUNX2 and FN1, which may be promising therapeutic targets for GC treatment. Further investigation is needed to elucidate the specific biological mechanisms of RUNX2 and FN1 in GC in the future, and the relationships among the other hub genes need to be clarified with additional research.
